# Weighted Gene Co-Expression Network Based on Transcriptomics: Unravelling the Differentiation Dynamics of 3T3-L1 Preadipocytes and the Regulatory Mechanism of Protopanaxatriol

**DOI:** 10.3390/ijms252212254

**Published:** 2024-11-14

**Authors:** Yaru Zhao, Xv Wang, Hongbo Teng, Tianyi Zhao, Wendyam Marie Christelle Nadembega, Xinhua Fan, Wenxin Zhang, Bowen Fan, Yuye Chi, Yan Zhao, Shuangli Liu

**Affiliations:** 1 College of Chinese Medicinal Materials, Jilin Agricultural University, Changchun 130117, China; zyr262396@163.com (Y.Z.); a1975227932@gmail.com (X.W.); 13614431169@163.com (H.T.); 13694379232@163.com (X.F.); jessica990125@163.com (W.Z.); 15103125937@163.com (B.F.); 15604306203@163.com (Y.C.); 2 International Joint Laboratory for Development of Animal and Plant Resources for Food and Medicine, Changchun 130118, China; tianyi63@163.com (T.Z.); christelle.nadembega@ujkz.bf (W.M.C.N.)

**Keywords:** obesity, protopanaxatriol, RNA sequencing, WGCNA, adipocyte differentiation, lipid accumulation

## Abstract

The intricate regulatory mechanisms governing adipocyte differentiation are pivotal in elucidating the complex pathophysiology underlying obesity. This study aims to explore the dynamic changes in gene expression during the differentiation of 3T3-L1 adipocytes using transcriptomics methods. Protopanaxatriol (PPT) significantly inhibited adipocyte differentiation. To uncover the molecular mechanisms, we conducted an extensive transcriptomic analysis of adipocytes throughout various differentiation stages, comparing gene expression profiles before and after PPT treatment. The construction of 16 co-expression modules was achieved using weighted gene co-expression network analysis (WGCNA). The 838 differentially expressed genes in the blue module were highly correlated with PPT treatment. Further analysis revealed that PIKfyve, STAT3, JAK1, CTTN, TYK2, JAK3, STAT2, STAT5b, SOCS3, and IRF9 were core genes closely associated with adipocyte differentiation. This discovery underscores the potential pivotal function of these ten genes in regulating adipocyte differentiation. This study elucidated that PPT, an active ingredient in ginseng, could reduce lipid accumulation by inhibiting the differentiation of adipocyte precursors through the negative regulation of genes such as PIKfyve, STAT3, and JAK1. Finally, molecular docking identified potential binding sites for PPT on PIKfyve and JAK1. This study provides potential drug targets for preventing obesity and related metabolic diseases.

## 1. Introduction

Obesity represents a significant global health concern arising from an imbalance between energy intake and expenditure. It is a considerable risk factor for a range of chronic diseases, including insulin (INS) resistance, type 2 diabetes, non-alcoholic fatty liver disease, and cardiovascular disease [1,2]. This condition represents a significant threat to public health and also places a considerable burden on the social economy [3]. It is, therefore, imperative that drugs and targets for the prevention and reversal of obesity and related metabolic diseases are developed as a matter of urgency.

Obesity often results from a sustained excess of energy intake over energy expenditure, leading to an increase in the size (hypertrophy) and number (hyperplasia) of adipocytes, which in turn contributes to an increase in adipose tissue mass. During this process, there is an increase in the synthesis and storage of triglycerides within adipocytes, which form lipid droplets [4]. The expansion of adipose tissue is mainly attributable to the uninterrupted differentiation of precursor cells into mature adipocytes [5,6]. The differentiation of adipocyte precursor cells into mature adipocytes is characterized by changes in cell phenotype [7]; this differentiation process is regulated by a network of transcription factors and regulatory factors [8]. Adipocyte precursor cells undergo various changes, including growth arrest, mitotic clonal expansion, and early and terminal differentiation [9]. In the terminal differentiation stage, preadipocytes undergo morphological changes, form lipid droplets, and eventually become mature adipocytes [10]. Nevertheless, the precise mechanisms of transcriptional regulation during the differentiation of adipocyte precursor cells remain unclear. It is, therefore, necessary to elucidate the regulatory mechanisms of differentiation at various stages of adipocyte precursor cell differentiation.

PIKfyve is a phosphatidylinositol 5-kinase that is pivotal in cellular metabolic regulation. It has been demonstrated that the inhibition of PIKfyve’s enzymatic activity affects insulin-mediated biological processes, including glucose transporter translocation, mitosis, and the differentiation of 3T3-L1 preadipocytes into mature adipocytes [11]. It has been demonstrated that the JAK2 and STAT3 pathways are also instrumental in the differentiation of preadipocytes [12]. The STAT3 pathway is activated at the outset of the differentiation process, where it plays a role in promoting the differentiation of adipocytes. This occurs via the JAK2/STAT3/C/EBPβ pathway [13]. The activation of STAT3 is accompanied by its translocation from the cytoplasm to the nucleus, where it directly regulates the transcription of differentiation-related genes such as C/EBPβ, thereby promoting the maturation process of adipocytes [14]. These insights indicate that PIKfyve and the JAK-STAT signaling pathway are pivotal in preventing and regulating adipocyte differentiation and regulatory mechanisms.

Traditional Chinese medicine has been widely used in the treatment of metabolic disorders such as obesity. The abundant natural compounds it contains can regulate processes related to adipogenesis. *Panax ginseng* is a species of Panax, which is part of the Araliaceae family. It is native to China, South Korea, and Japan, where it is widely distributed. In China, the species is predominantly found in the Changbai Mountains and the Greater Khingan Range. It is a plant that can be consumed and has medicinal properties. Given its favorable and diverse biological activities and safety profile, the prospects for its development are promising [15]. Ginsenosides represent the primary active constituents of ginseng and have been demonstrated to exert a range of pharmacological effects, including anti-obesity, anti-inflammatory, and anti-diabetic properties [16]. Rg3 has been shown to inhibit NF-κB-mediated macrophage activation through the PPARγ target, thereby improving obesity-induced insulin resistance [17]. Ginsenoside Rg1 has been demonstrated to activate brown fat by regulating the intestinal flora and bile acid profile, thereby improving high-fat diet-induced obesity in mice [18]. Nevertheless, ginsenosides’ regulatory and mechanical effects on the differentiation of preadipocytes remain unreported.

This study aimed to evaluate the inhibitory effects of various ginsenosides on the differentiation of preadipocytes. Cell activity screening determined that PPT has a notable inhibitory impact on preadipocyte differentiation. We conducted transcriptome sequencing on preadipocytes at various differentiation stages before and after PPT treatment. The WGCNA method was employed to construct co-expression modules, identifying modules related to genes associated with adipocyte differentiation stages. By integrating these modules with differential gene expression analysis, we sought to identify essential hub genes within the core modules to discover critical genes that influence various stages of preadipocyte differentiation.

It is of particular significance that our findings indicate that the active ingredient PPT in ginseng may exert a negative regulatory effect on the differentiation process of preadipocytes through the modulation of genes such as PIKfyve, STAT3, and JAK1, thereby reducing lipid accumulation. This study broadens the mechanisms and targets for anti-obesity and provides a new, potentially effective drug for preventing obesity and related metabolic diseases.

## 2. Results

### 2.1. The Effect of Ginsenosides on the Viability of 3T3-L1 Preadipocytes (Undifferentiated Cells) and Mature Adipocytes (Differentiated Cells)

**Figure 1:**
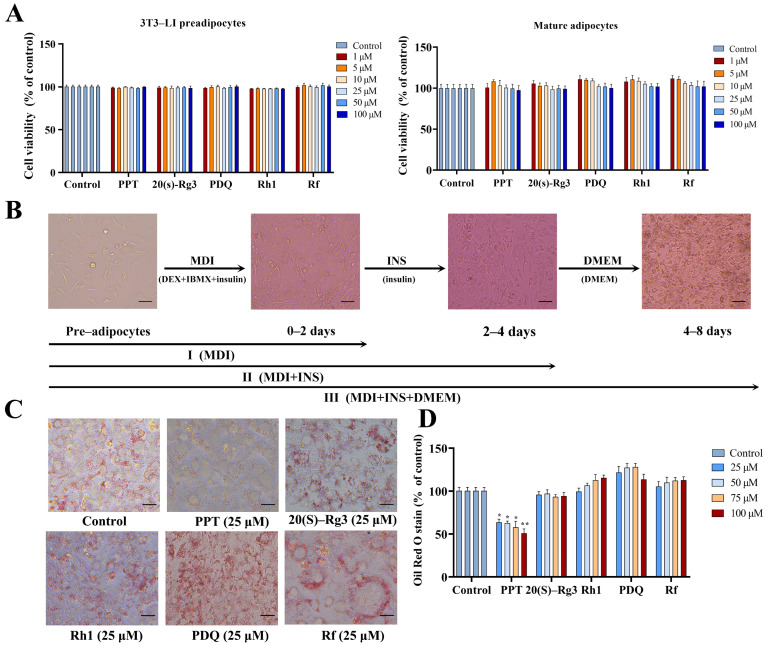
A schematic representation of 3T3-L1 preadipocyte differentiation and the inhibitory effect of PPT on 3T3-L1 preadipocyte differentiation. (**A**) The effect of different concentrations of ginsenosides on the viability of 3T3-L1 preadipocytes (undifferentiated cells) and mature adipocytes (differentiated cells). (**B**) A schematic representation of different stages of 3T3-L1 preadipocyte differentiation. (**C**) Oil Red O staining images of 3T3-L1 cells after eight days of differentiation. (**D**) Quantitative results of Oil Red O staining. Scale bar: 150 µm. The data are expressed as mean ± standard deviation. (*) *p* < 0.05, (**) *p* < 0.01 compared to the control.

As shown in Figure 1A, after treatment with different concentrations of ginsenosides (1, 5, 10, 25, 50, 100 μM) on 3T3-L1 adipocyte precursor cells, it was found that ginsenosides PPT, 20 (s)-Rg3, PDQ, Rh1, and Rf had no significant effect on the viability of 3T3-L1 adipocyte precursor cells. As shown in Figure 1B, after treatment with different concentrations of ginsenosides on 3T3-L1 mature adipocytes, it was found to have no significant effect on the viability of 3T3-L1 mature adipocytes. From this, it can be concluded that ginsenosides have no significant cytotoxic effect on 3T3-L1 adipocyte precursor cells (undifferentiated cells) and mature adipocytes (differentiated cells) within the concentration range of 0–100 µM. Subsequent induction differentiation experiments were performed using this concentration.

### 2.2. Morphological Changes of 3T3-L1 Preadipocytes at Different Stages of Differentiation

We used the “cocktail” method of adipogenic induction to induce the differentiation of 3T3-L1 preadipocytes. These are representative images of the 3T3-L1 preadipocytes at different stages: undifferentiated preadipocytes, 2 days of differentiation, 4 days of differentiation, and 8 days of differentiation, labelled as C0, stage I (C1), stage II (C2), and stage III (C3) (Figure 1B). Through three stages of induction, the 3T3-L1 preadipocytes have differentiated into mature adipocytes.

### 2.3. Ginsenosides Inhibit Lipid Accumulation and the Degree of Differentiation of 3T3-L1 Preadipocytes

Based on the previous cell viability assay, we selected these different ginsenosides to act on the entire process of differentiation of 3T3-L1 preadipocytes. Once differentiation was complete, Oil Red O staining was used to detect the effect of ginsenosides on adipocyte differentiation. As shown in Figure 1C, after the control group cells were cultured in an environment containing a differentiation medium for eight days, a solid staining area was shown, indicating that the preadipocytes had differentiated into mature adipocytes containing many lipid droplets. However, with the addition of different ginsenosides, the results showed that only in the PPT treatment group was the Oil Red O staining area in the adipocytes significantly reduced, indicating that PPT can inhibit the formation of lipid droplets and thus inhibit the differentiation of 3T3-L1 preadipocytes. Obesity is characterized by increased size (hypertrophy) and number (hyperplasia) of adipocytes, resulting in increased adipose tissue mass. This is caused by the hypertrophy of adipocytes and the differentiation of preadipocytes into mature adipocytes. We measured lipid accumulation in adipocytes after eight days of differentiation to evaluate the inhibitory effects of different ginsenosides on adipocyte differentiation. As shown in Figure 1D, within the 25–100 μM dosage range, most ginsenosides did not show significant inhibitory effects compared to the control group. However, PPT significantly reduced lipid accumulation in 3T3-L1 adipocytes at all concentrations, effectively inhibiting adipocyte differentiation. In particular, at a concentration of 100 μM, lipid accumulation in the PPT-treated group was only 50.8% of that in the control group. This indicates that PPT significantly inhibited the accumulation of lipid droplets in 3T3-L1 cells, effectively suppressing the differentiation of preadipocytes into mature adipocytes. We chose PPT as the subject of our research to further investigate its mechanism of action at different stages of preadipocyte differentiation. We designated the preadipocytes as group C0, the three stages of the control group as C1, C2, and C3, and the three stages of the PPT treatment group as T1, T2, and T3. We collected RNA from each of these seven groups for subsequent transcriptomic analysis.

### 2.4. Transcriptional Regulation During Differentiation of 3T3-L1 Preadipocytes

**Figure 2:**
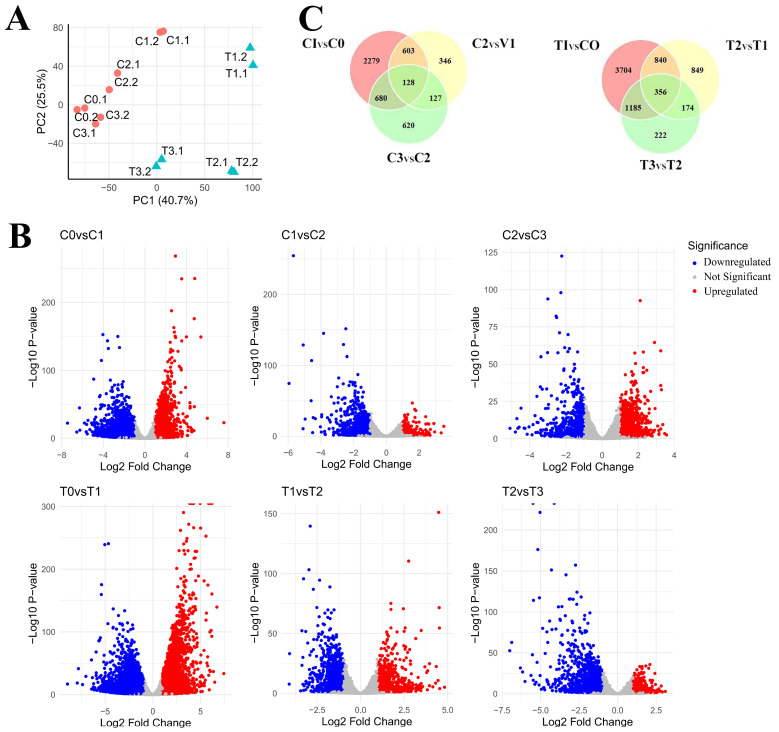
Differentially expressed genes in 3T3-L1 preadipocytes at three different stages of differentiation. (**A**) The principal component analysis method was used to evaluate the gene expression levels of 3T3-L1 preadipocytes at different differentiation stages (C1, C2, C3, T1, T2, T3). Red circles represent samples of control adipocytes at three different stages of differentiation, and green triangles represent samples of PPT-treated adipocytes at three different stages of differentiation. (**B**) Volcano plots of samples with differential mRNA expression. Log2 (fold change) is plotted as abscissa and log10 (adjusted *p*-value) as ordinate. The representative distribution of gene upregulation or downregulation between each of the two stages is shown in the volcano plot. Significantly, upregulated genes are shown in red, and downregulated genes in blue. (**C**) A Venn diagram showing the number of unique or shared DEGs (differentially expressed genes) in the control and PPT treatment groups.

Using RNA-seq technology, we performed a detailed analysis of the transcriptomic data of 3T3-L1 adipocytes at three different differentiation stages under normal conditions and PPT treatment. Principal component analysis (PCA) evaluated the distribution of the total samples on two principal components (PC1 axis and PC2 axis). The correlation of gene expression levels in different samples is an essential criterion for testing experimental reliability and the rationality of sample selection. Analysis of the PCA results shows that two samples of 3T3L-1 adipocytes in the control group, C0.1 and C0.2, C1.1 and C1.2, C2.1 and C2.2, C3.1 and C3.2, clustered together. In the PPT treatment group, two samples of 3T3L-1 adipocytes (T1.1 and T1.2, T2.1 and T2.2, T3.1 and T3.2) cluster together. The total sample data show that the 3T3-L1 adipocytes in the PPT treatment group and the control group can be divided into three different stages of differentiation. There are two samples in each group, and each group’s distribution can be clearly distinguished on the plotted PC1 (40.7%) and PC2 (25.5%) coordinate axes. (Figure 2A). RNA-seq results revealed the dynamic changes in gene expression of 3T3-L1 preadipocytes in both the control and PPT treatment groups. In the control group, stage I showed that 1801 genes upregulated and 1440 genes downregulated; stage II showed that 715 genes upregulated and 172 genes downregulated; stage III showed that 451 genes upregulated and 706 genes downregulated. These results suggest that the formation and differentiation of adipocytes is a highly complex and finely regulated biological process that marks the transition from preadipocytes to mature adipocytes. Accordingly, during the differentiation process of PPT-treated 3T3-L1 preadipocytes, the RNA-seq results showed that in stage I, 2620 genes were upregulated and 2998 genes were downregulated; in stage II, 982 genes were upregulated and 818 genes were downregulated; in stage III, 1048 genes were upregulated, and 472 genes were downregulated (Figure 2B). The PPT treatment group showed many differentially expressed genes at each stage of differentiation, particularly at stage I, where the number of differentially expressed genes was highest. This further confirms the significant effect of PPT on adipocyte differentiation. To gain a deeper understanding of the dynamic changes in gene expression during adipocyte differentiation and the impact of PPT on this process, we compared the differentially expressed genes at different stages in the control group, including C1 vs. C0 (stage I), C2 vs. C1 (stage II), and C3 vs. C2 (stage III). At the same time, we performed a comparative analysis between the PPT treatment group and the control group, including T1 vs. C0 (stage I), T2 vs. T1 (stage II), and T3 vs. T2 (stage III). This approach allowed us to identify stage-dependent differentially expressed genes involved in lipid accumulation and adipocyte differentiation, thus providing a more precise understanding of the effects of PPT on different stages of adipocyte differentiation. Venn analysis showed that 3T3-L1 adipose progenitor cells in the PPT-treated and control groups exhibited distinct gene expression changes throughout the differentiation process. The number of uniquely or co-differentially expressed genes in the PPT-treated group (T1, T2, T3) and the control group (C1, C2, C3) was analyzed in Figure 2C. The PPT-treated group had more differentially expressed genes at the initial stage (T1vsC0), while the control group had fewer differentially expressed genes at the initial stage (C1 vs. C0). The number of genes co-differentially expressed at different stages was higher in the PPT-treated group (356) than in the control group (128), suggesting that the PPT-treated group may have resulted in more sustained gene expression changes.

### 2.5. Construction of Weighted Gene Co-Expression Modules

**Figure 3:**
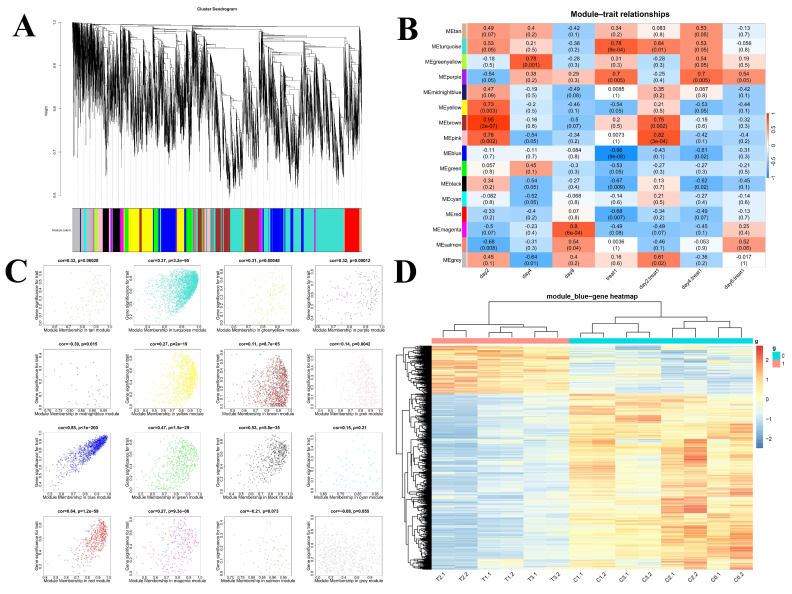
WGCNA. (**A**) The partitioning of gene modules. This figure illustrates the clustering of genes, and the division of gene modules is based on this result. The branches of the same color were assigned to the same gene module. (**B**) Associations between modules and traits. Panel A illustrates the correlation between gene module and sample information, with the *x*-axis representing sample information and the *y*-axis representing each gene module. The color intensity indicates the strength of the correlation, with red representing a positive correlation and blue representing a negative correlation. The *p*-value, which represents the significance value, is provided in brackets. (**C**) Scatterplots of gene significance (GS) (*y*-axis) versus module membership (MM) (*x*-axis) for sixteen modules. This figure illustrates the degree of significance attributed to the genes within the sixteen modules. (**D**) The clustering heatmap demonstrates the expression of 838 genes within the blue module.

To further elucidate the effect of PPT on the differentiation process of 3T3-L1 preadipocytes, we constructed a differential gene co-expression network using the WGCNA method. First, the gene expression data from all groups (C0, C1–C3, T1–T3) were normalized, and genes with low expression levels or a high proportion of missing values were excluded to ensure data quality and reliability. Using the dynamic tree-cutting method, we identified sixteen gene modules through gene clustering analysis. Each module consists of a set of genes that show high co-expression in all samples. Figure 3A shows the gene dendrogram, where each branch represents a gene, and different colors indicate different gene modules. These modules represent groups of genes that may play critical roles in the differentiation process of preadipocytes under the action of PPT.

### 2.6. Identifying the Most Relevant Module

To gain a deeper understanding of the intricate relationship between gene modules and the various sample treatment groups, we employed multi-dimensional analysis and visualization techniques to elucidate the influence of PPT on the adipocyte differentiation process. The correlation between module eigengenes (MEs) and different treatment groups was calculated, and a module–trait relationship heatmap was generated (Figure 3B). Following a comprehensive analysis, we were able to ascertain that 16 modules were significantly associated with adipocyte differentiation traits, thereby establishing a robust foundation for subsequent in-depth research. As shown in Supplementary Figure S1A, the box plots provide a clear visualization of the distribution of expression of genes characterizing each module across all samples. This allows for an intuitive comparison of the expression level differences between module eigengenes across different sample groups. Subsequently, a scatter plot (Figure 3C) was constructed to illustrate the correlation between module eigengenes and adipocyte differentiation traits. Notably, the blue module exhibits the highest correlation with adipocyte differentiation (r = 0.96, *p* = 9 × 10^−8^), suggesting that the genes in the blue module may play a pivotal role in the process of PPT affecting adipocyte differentiation.

Subsequently, a cluster heatmap analysis was conducted on the 838 genes of the blue module (3D). The expression of differential genes in adipocytes at different differentiation stages in the control and administration groups can be more clearly discerned. Upon examination of the horizontal samples, it was observed that the preadipocytes (C0) and the two biological replicate samples of the adipocytes in the control group (C1, C2, C3) formed distinct clusters. The two biological replicate samples of the adipocytes in the three differentiation stages (T1), (T2), and (T3) of the PPT administration group formed clusters, indicating a high degree of correlation between the repeated samples. A longitudinal gene clustering analysis was performed. The gene expression profiles were divided into two distinct clusters, representing the successful clustering of the upregulated and downregulated genes. Significant discrepancies in gene expression profiles were discerned between the preadipocytes (C0) and the cells belonging to the control group (C1, C2, C3). Moreover, the gene expression profiles of the preadipocytes (C0) and the cells of the PPT group (T1, T2, T3) also exhibited significant divergences. The genes that were upregulated and downregulated, respectively, showed a high degree of correlation and considerable separation.

### 2.7. A Functional Enrichment Analysis Was Conducted on the Genes Within the Blue Module

**Figure 4:**
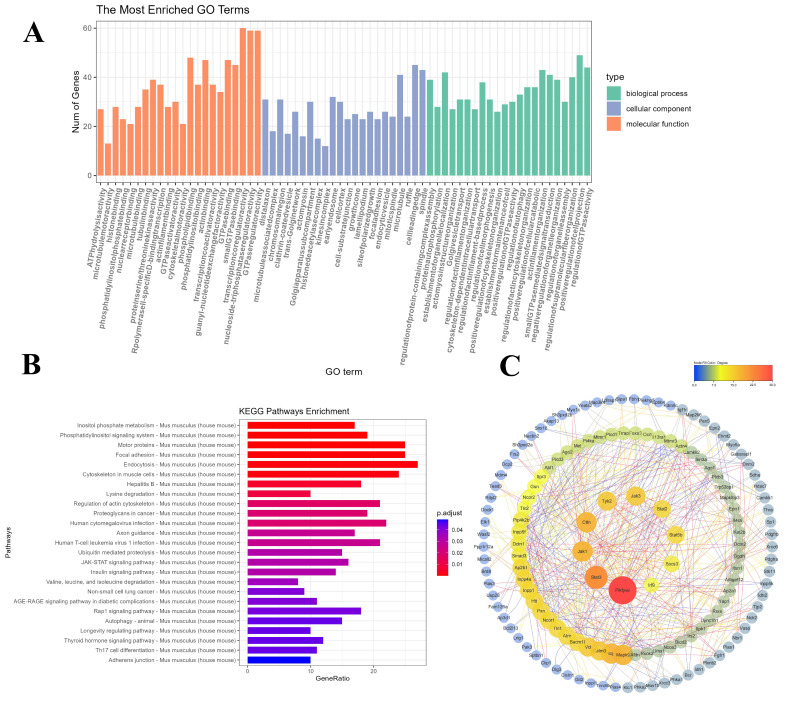
GO and KEGG functional enrichment analyses of differentially expressed genes in the blue module. (**A**) The comparison of GO enrichment. It shows the top 20 significantly enriched GO terms, including biological process, cellular component, and molecular function. (**B**) KEGG pathway enrichment. It shows the top 20 significantly enriched KEGG pathways. (**C**) Protein–protein interaction network of blue module genes. The edge represents interactions between genes. A degree describes the importance of the protein nodes in the network. The top 10 genes with the highest degree values were found using CytoHubba_MCC, and the depth of the color corresponded to the weighted score.

To gain insight into the functional characteristics of genes in the blue module and their potential roles in the adipocyte differentiation process, a comprehensive Gene Ontology (GO) analysis and a Kyoto Encyclopedia of Genes and Genomes (KEGG) pathway analysis was conducted. As illustrated in Figure 4A, the outcomes of the GO analysis suggest that these enriched genes are predominantly involved in biological processes about cell division, intracellular signal transduction, and transcriptional regulation. These processes are closely related to the differentiation and lipid accumulation of adipocytes, indicating that these genes may play a pivotal role in the development and function of adipocytes. A further KEGG analysis (Figure 4B) reveals the significantly enriched biological pathways of the blue module genes, including inositol phosphate metabolism, the phosphatidylinositol signaling system, the JAK-STAT signaling pathway, insulin signaling transduction, and so forth. These pathways play a pivotal role in cellular metabolism, signal transduction, and growth regulation and may serve as the primary mediators of the inhibitory effect of PPT on adipocyte differentiation.

### 2.8. PPI (Protein–Protein Interaction) Network Analysis of the Hub Genes

As illustrated in Figure 4C, a protein–protein interaction (PPI) network was constructed among the overlapping genes using the STRING database. The MCC plugin allowed the CytoHubba algorithm to identify the key regulatory elements within the network and select the ten core genes with the highest scores. The following core genes have been identified: PIKfyve, STAT3, JAK1, CTTN, TYK2, JAK3, STAT2, STAT5b, SOCS3, and IRF9. Based on the evidence provided by these highly connected hub genes, we hypothesize that they may play a central role in the process of PPT inhibiting adipocyte differentiation. It is conceivable that these genes act synergistically to regulate several key signaling pathways and cellular processes, ultimately inhibiting 3T3-L1 preadipocyte differentiation.

### 2.9. Expression and Validation of Core Genes in 3T3-L1 Preadipocytes at Different Differentiation Stages

**Figure 5:**
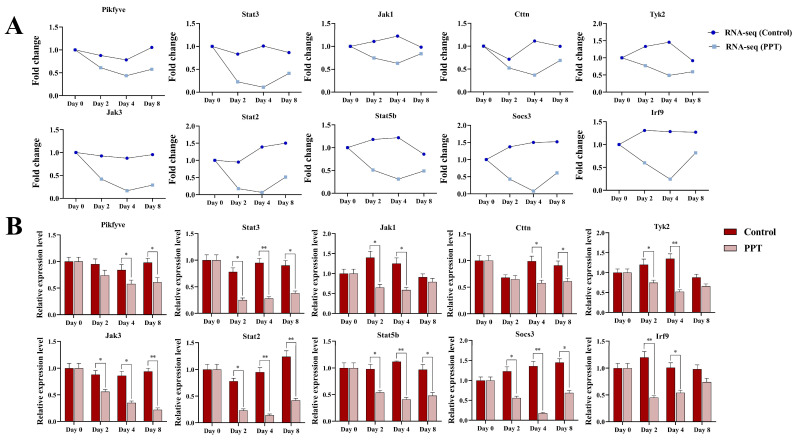
Expression and verification of core genes in the blue module. (**A**) Expression trend of the core genes in the blue module. (**B**) Relative mRNA expression levels of core genes in the blue module. (*) *p* < 0.05, (**) *p* < 0.01 compared to the control.

By analyzing the gene network in the blue module, we identified the top ten core genes with the highest scores in the PPI network, including PIKfyve, STAT3, JAK1, CTTN, TYK2, JAK3, STAT2, STAT5b, SOCS3, and IRF9. Of these, STAT3, JAK1, TYK2, JAK3, STAT2, STAT5b, SOCS3, and IRF9 were found to be significantly enriched in the JAK-STAT signaling pathway. As illustrated in Figure 5A, the expression levels of these genes in the PPT treatment group exhibited a downward trend relative to the control group as the differentiation days of the preadipocytes increased. The expression pattern of these differentially expressed genes provides further evidence to support the hypothesis that PPT inhibits preadipocyte differentiation by negatively regulating these genes. These core genes involve several critical biological processes, including lipid metabolism, cell proliferation, and differentiation regulation. The three nodes with the highest number of connections are PIKfyve, STAT3, and JAK1, which suggests that PPT’s inhibition of 3T3-L1 preadipocyte differentiation may be primarily related to inositol phosphate metabolism and the JAK-STAT signaling pathway [19]. Subsequently, we conducted RT-qPCR to validate the expression levels of the genes mentioned above, thereby verifying the differential expression observed in the RNA-Seq analysis.

As illustrated in Figure 5B, comparing the RNA-Seq data with the qRT-PCR results reveals a comparable trend in the expression changes of the selected core genes. This indicates that the differentially expressed genes identified using transcriptome data are effective and reliable. Incorporating PPT may markedly prolong the process of hormone-induced adipogenesis during the differentiation of 3T3-L1 preadipocytes into mature adipocytes. This may be related to inhibiting the lipid kinase activity of PIKfyve. Alternatively, it may be related to modulation of the JAK-STAT pathway.

### 2.10. Molecular Modeling

**Figure 6:**
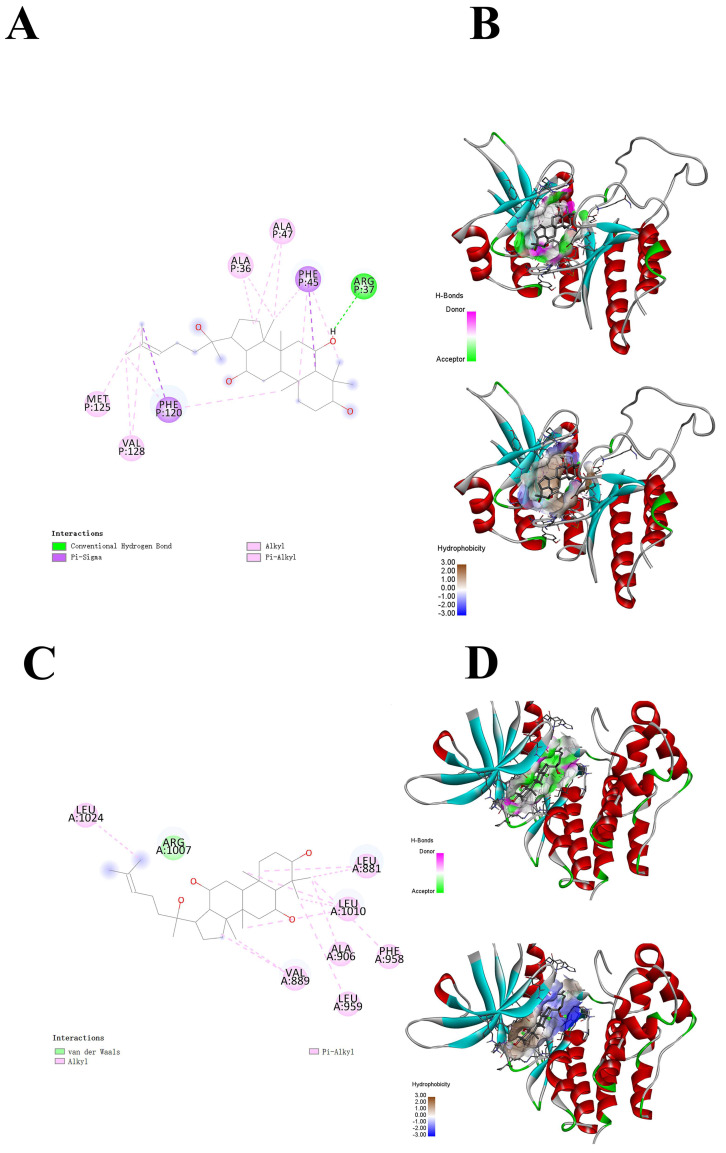
Molecular docking of PPT with PIKfyve and JAK1. (**A**) The planar binding conformation of PPT and the PIKfyve protein (interaction forces with protein amino acid residues). (**B**) Hydrogen bonds and hydrophobic interactions between PPT and the protein domain of PIKfyve. (**C**) The planar binding conformation of PPT and the JAK1 protein (interaction forces with protein amino acid residues). (**D**) Hydrogen bonds and hydrophobic interactions between PPT and the JAK1 protein domain.

In this study, two proteins with relatively high docking scores were selected for research to investigate the mechanism of action of PPT further. Molecular docking was carried out using Autodock Vina. The docking score of PIKfyve with PPT was −9.4 kcal/mol, and that with JAK1 was −9.3 kcal/mol, both of which were less than −5 kcal/mol, indicating that PPT had binding solid affinity with both of these two proteins. Figure 6A shows the interactions of PPT with multiple amino acid residues of the PIKfyve protein, including ALA36, ALA47, PHE45, ARG37, MET125, PHE120, VAL128, etc. Figure 6B displays the three-dimensional structure of the PIKfyve protein, with PPT embedded inside the binding pocket of PIKfyve and surrounded by the surrounding protein structures. The binding of PPT to PIKfyve involves multiple non-covalent interactions, including hydrogen bonds, π-σ, alkyl, π-alkyl interactions, and hydrophobic interactions. These interactions work together to promote the high complementarity and specific binding of PPT to the binding pocket of PIKfyve.

As shown in Figure 6C, the interactions between PPT and several amino acid residues of the JAK1 protein include LEU A:1024, ARG A:1007, LEU A:881, LEU A:1010, ALA A:906, PHE A:958, VAL A:889, and LEU A:959, etc. These interactions, including van der Waals forces, alkyl interactions, and π-alkyl interactions, collectively promote the specific binding of PPT to the JAK1 protein. The binding site of PPT is mainly located in the hydrophobic region of the JAK1 protein, which helps it to form a stable interaction with the hydrophobic pocket of the protein. In addition, the hydrogen bond network between protopanaxatriol and the JAK1 protein plays a crucial role in maintaining the binding stability. Molecular docking reveals the potential binding region of PPT in JAK1. This further confirms that PPT has a superior spatial conformation due to its appropriate molecular weight and spatial distribution. Therefore, it can bind well to the active pockets of PIKfyve and JAK1.

## 3. Discussion

The primary characteristic of obesity is the enlargement of adipose tissue, which occurs through an increase in both the size (hypertrophy) and number (hyperplasia) of adipocytes [20]. These two processes are highly dependent on the regulation of adipocyte differentiation. Numerous studies have shown that inhibiting adipocyte proliferation can effectively modulate the adipocyte differentiation process [21]. The 3T3-L1 cell line is a well-established model for studying adipocyte differentiation. This cell line is derived from preadipocytes and displays a morphology comparable to fibroblast-like cells. When subjected to appropriate induction conditions, 3T3-L1 preadipocytes can undergo effective differentiation into mature adipocytes, which are characterized by numerous lipid droplets (Figure 1C) The adipocyte differentiation process is characterized by high complexity and precise regulation. The differentiation of preadipocytes into mature adipocytes can be divided into four key stages: growth arrest, mitotic clonal expansion, early differentiation, and terminal differentiation [8]. In 3T3-L1 cells, contact inhibition between cells results in growth arrest. Preadipocytes that have undergone confluence enter the stage of mitotic clonal expansion when stimulated by adipogenic inducers, including fetal bovine serum (FBS), dexamethasone (DEX), 3-isobutyl-1-methylxanthine (IBMX), and insulin (INS). Many lipid droplets are formed during the terminal differentiation process, and preadipocytes ultimately differentiate into mature adipocytes [22].

Currently, traditional Chinese medicine is a widely utilized approach in the management of metabolic disorders, including obesity. Ginseng is a plant that can be consumed and used for medicinal purposes. Given its favorable and diverse biological activities and safety profile, the outlook for its future development is promising [15]. Ginsenosides, the primary active components of ginseng, have been demonstrated to exert a range of pharmacological effects, including the reduction of obesity [23], the improvement of insulin resistance [24], and the promotion of anti-diabetic properties [25]. The initial screening aimed to evaluate the inhibitory effects of various ginsenosides on the differentiation of preadipocytes. To gain further insight into the anti-obesity effects of ginsenosides, a systematic screening of multiple ginsenosides was conducted, focusing on their ability to inhibit preadipocyte differentiation. The findings revealed that PPT exhibited the most pronounced effect, effectively inhibiting the differentiation of preadipocytes.

The differentiation of preadipocytes is regulated by a complex network of multiple genes that interact with one another. In traditional one-dimensional studies, identifying the essential genes and their primary mechanisms of action often presents a significant challenge. However, WGCNA can effectively utilize phenotypic information to transform associations between thousands of genes and phenotypes into associations between multiple gene sets and phenotypes, thereby effectively reflecting gene interactions. To gain further insight into the effects of PPT on preadipocyte differentiation, we conducted a comprehensive transcriptome sequencing analysis covering all differentiation stages before and after PPT treatment. The WGCNA analysis identified sixteen gene modules, each composed of gene sets that are highly co-expressed across all samples. The study revealed that the blue module exhibited the highest correlation with PPT treatment (r = 0.96, *p* = 9 × 10^−8^), thus providing a crucial foundation for identifying core genes.

We conducted GO functional and KEGG pathway analyses to gain further insight into the mechanisms of action of core genes in the blue module during preadipocyte differentiation. The GO analysis demonstrated that the biological processes associated with these enriched genes are related to cell division, intracellular signal transduction, and transcriptional regulation. These processes are closely linked to adipocyte differentiation and lipid accumulation. The KEGG enrichment analysis revealed that the functions of these pathways are primarily concentrated in crucial biological pathways, including inositol phosphate metabolism, the phosphatidylinositol signaling system, the JAK-STAT signaling pathway, and the insulin signaling pathway.

Subsequently, the ten core genes with the highest scores from the PPI network were selected, with PIKfyve being the node with the most connections. PIKfyve is expressed at varying levels in different tissues and cells, with the highest expression levels observed in the brain and adipose tissue. PIKfyve and its enzymatic products demonstrate pleiotropy, regulating many cellular processes, including membrane trafficking, stress- or hormone-induced signal transduction, ion channel activity, cytoskeletal dynamics, nuclear transport, gene transcription, and cell cycle progression [26]. PIKfyve is a crucial lipid kinase that plays a vital role in the dynamics of intracellular membranes and signal transduction. The enzymatic activity of PIKfyve is critical for the insulin-mediated translocation of GLUT4. It has been demonstrated that the inhibition of PIKfyve activity can markedly diminish the translocation of GLUT4 to the plasma membrane in insulin-induced adipocytes, consequently impairing the cell’s capacity to absorb glucose [27]. Furthermore, studies have indicated that PIKfyve enzymatic activity not only participates in insulin signal transduction and glucose metabolism but also plays an active role in the phenotypic transformation of fibroblasts into adipocytes. The conversion process is typically induced by adipogenic hormones, including insulin and glucocorticoids [28]. The inhibition of PIKfyve enzymatic activity has been demonstrated to diminish several insulin-stimulated biological effects, including glucose transport, glucose transporter translocation, and mitosis, as well as the differentiation of 3T3-L1 fibroblasts into adipocytes. The results of this study suggest that PIKfyve enzymatic activity may also exert a positive regulatory effect on the process of adipogenesis [11]. The results demonstrate that 3T3-L1 preadipocytes enter the mitotic clonal expansion phase in the regular differentiation group influenced by adipogenic inducers and enter the mitotic clonal expansion phase in the regular differentiation group. Furthermore, during terminal differentiation, extensive accumulation of lipid droplets is observed as the cells differentiate into mature adipocytes. Concurrently, following PPT treatment, it was observed that 3T3-L1 preadipocytes exhibited a delay in the hormone-induced differentiation to the adipocyte phenotype. These findings suggest that PPT may act as an effective inhibitor of PIKfyve kinase, thereby reducing hormone-induced adipogenesis and slowing down the process of preadipocyte differentiation.

Furthermore, our research indicates that the JAK-STAT signaling pathway plays a crucial role in the processes of adipogenesis. Metabolic regulation findings suggest that STAT3, JAK1, TYK2, JAK3, STAT2, STAT5b, SOCS3, and IRF9 are significantly enriched in the JAK-STAT signal, suggesting that these genes may play a pivotal role in the process of adipocyte differentiation. The functions of the JAK-STAT signaling pathway include the regulation of adipocyte differentiation, insulin action, and the transcriptional regulation of genes involved in lipid and glucose metabolism [14]. The JAK and STAT family of proteins that can be detected in adipocytes include JAK1/2 (JAKs) and STAT1/3/5/6 (STATs). It should be noted that JAKs do not act independently on adipocytes. The inhibitory effect on adipocyte differentiation is produced via STATs [19]. Moreover, research has demonstrated that JAK1 may regulate mesenchymal stem cell (MSCs) differentiation into adipocytes via activating the STATs signaling pathway [29]. In the initial stages of adipogenesis, the JAK2/STAT3 pathway is activated, which has been demonstrated to potentially promote adipocyte differentiation by regulating the transcription of C/EBPβ [13,30]. The silencing of STAT3 has been shown to play a crucial role in ensuring the adequate expression of essential transcription factors for the process of adipogenesis. It has been demonstrated that cryptotanshinone inhibits the process of adipogenesis by preventing the phosphorylation of STAT3 in adipocytes [31]. It has been shown that the STAT5A and STAT5B proteins are markedly induced during the differentiation of adipose cells. Furthermore, their expression is closely correlated with the accumulation of lipids and the expression of the peroxisome proliferator-activated receptor gamma (PPARγ) [32]. Subsequent in vivo studies have demonstrated that the deficiency of STAT5a, STAT5b, or both results in a substantial reduction in adipose pad size in mice, indicating that STAT5 proteins play a pivotal role in adipose tissue formation [33]. Furthermore, TYK2, located upstream of the STAT pathway, plays a role in adipose tissue differentiation. Oleanolic acid may represent an efficacious anti-obesity strategy through the selective blockade of the Tyk-STAT pathway. Furthermore, TYK2 has been demonstrated to play a role in regulating energy balance. It has been shown that the reduction of TYK2 and STAT1 in adipose tissue promotes the expression of PPARγ and FAS, thereby regulating lipid metabolism [34]. The SOCS protein family, which functions as a negative regulator of cytokine signaling, plays a pivotal role in modulating crucial biological processes [35]. Cytokines regulate cellular proliferation, differentiation, and apoptosis processes by activating plasma membrane receptors and recruiting the JAK-STAT pathway, thereby controlling the transcription of multiple genes [36]. It is noteworthy that SOCS3 expression is elevated in obesity. The development of drugs that inhibit SOCS3 activity or expression may, therefore, represent a potential strategy for treating type 2 diabetes, obesity, and other metabolic disorders [37]. In obese rodents, SOCS3 is upregulated in the hypothalamus [38], liver [39], skeletal muscle [40], and adipose tissue [41]. In vitro studies also showed that overexpression of SOCS3 in adipocytes can cause local insulin resistance in adipocytes but not enough to cause systemic insulin resistance [42]. IRF9 plays a role in the regulation of infectious diseases and autoimmune disorders. Additionally, its dysfunction is linked to a range of metabolic disturbances, including insulin resistance, obesity, and cardiovascular diseases [43]. The results of our research indicate that these genes, which are significantly enriched in the JAK-STAT signaling pathway, play a pivotal role in the process of adipocyte differentiation. Notably, these genes are significantly downregulated in the PPT group compared to the control group. This finding suggests that PPT may impede the differentiation of 3T3-L1 adipocytes by inhibiting essential genes within the JAK-STAT signaling pathway.

In conclusion, in this study, by modeling the differentiation dynamics of 3T3-L1 preadipocytes, we found that protopanaxatriol (PPT) significantly inhibited the differentiation of adipocyte precursors and reduced lipid accumulation; weighted gene co-expression network analysis based on transcriptomics indicated that PPT may regulate adipocyte differentiation and reduce lipid accumulation by negatively regulating the expression of genes such as PIKfyve, STAT3, and JAK1. The potential binding sites of PPT with PIKfyve and JAK1 were further identified by molecular docking. Considering the limitations of cellular experiments and the functionality of fatty tissue, subsequent studies will verify the accuracy of the above experimental results by using whole animal experiments to investigate the efficacy and safety of PPT as a potential therapeutic drug for obesity, which will provide necessary theoretical and experimental bases for the development of innovative therapeutic solutions and relevant pharmaceutical agents for obesity and related metabolic diseases.

## 4. Methods

### 4.1. Materials

The 3T3-L1 preadipocytes were purchased from Wuhan Punuosai Life Science and Technology Co., Ltd., Wuhan, China; all cell line s were tested for STR identification and mycoplasma contamination at the time of purchase; Ginsenosides 20(S)-Rg3, Rf, Rh1, Pseudo-ginsenoside DQ (PDQ), and protopanaxatriol (PPT) were purchased from Shanghai Yuanye Biotechnology Co., Ltd., Shanghai, China; the purities of all are over 98%; Recombinant human insulin (INS) was purchased from Wuhan Punuosai Life Science and Technology Co., Ltd., Wuhan, China; DMEM (Dulbecco’s modified Eagle’s medium) medium was purchased from Beijing HyClone Biological Technology Co., Ltd., Beijing, China; Dexamethasone (DEX) and 3-isobutyl-1-methylxanthine (IBMX) were purchased from Shanghai Yuanye Biotechnology Co., Ltd., Shanghai, China; Cell Counting Kit-8 (CCK-8) was purchased from Good Laboratory Practice Bioscience, Montclair, USA; trypsin digestion solution and penicillin–streptomycin mixture were purchased from Beijing Solarbio Science & Technology Co., Ltd., Beijing, China; the Oil Red O staining kit was purchased from Beyotime Biotechnology Co., Ltd., Shanghai, China; TRIzol reagent was purchased from Solarbio Biotechnology Co., Ltd., Beijing, China.

### 4.2. Cell Culture and Differentiation

As previously described, the differentiation of 3T3-L1 preadipocytes was induced [44]. 3T3-L1 preadipocytes were cultured in 6-well plates at 37 °C and 5% CO_2_ until confluence, followed by an additional two days of incubation (day 0). Preadipocyte differentiation was induced for two days (referred to as phase I) using a differentiation medium (MDI) containing 0.5 mM IBMX, one μM DEX, and 10 μg/mL INS in DMEM supplemented with 10% FBS and 1% penicillin/streptomycin. The cells were then cultured for two days (phase II) in a maintenance medium containing insulin (10 μg/mL) in DMEM with 10% FBS and 1% penicillin/streptomycin. The medium was then changed to a standard complete medium. After eight days of induced differentiation (phase III), the preadipocytes were fully differentiated into mature adipocytes. Different ginsenosides were added at the beginning of the differentiation stage of 3T3-L1 preadipocytes, allowing the compounds to act on various phases of 3T3-L1 preadipocyte differentiation. Drug treatment dose design: 3T3-L1 preadipocytes were treated with a concentration gradient of 0, 25, 50, 75, and 100 μM to observe their differentiation.

### 4.3. CCK-8 Assay for Cell Activity in Each Group

Cell viability was assessed using the CCK-8 assay. 3T3-L1 preadipocytes (undifferentiated cells) and mature adipocytes (differentiated cells) were seeded in 96-well plates (5 × 10^3^ cells/well) and incubated overnight. The cells were then treated with different concentrations of ginsenoside for 24 h. Subsequently, 10 μL CCK-8 reagent was added to each well and incubated for 2 h. Cell viability was assessed by measuring the OD value of each group at 450 nm using a microplate reader (SpectraMax 190; Molecular Devices Co., Ltd., Shanghai, China).

### 4.4. Oil Red O Staining and Lipid Measurement

Lipid droplets in adipocytes were detected using an Oil Red O staining kit [45]. Briefly, 3T3-L1 preadipocytes were fixed with 4% paraformaldehyde for 1 h at room temperature and washed with 60% isopropanol. The cells were then stained with Oil Red O solution for 10 min. Images of the stained adipocytes were taken under a microscope. Finally, lipid droplets were dissolved in 60% isopropanol, and the lipid content was quantified by measuring the absorbance at 490 nm using a microplate reader.

### 4.5. RNA Extraction and Sequencing

3T3-L1 preadipocytes at different stages of differentiation were treated with or without PPT (25 μM) for 24 h. RNA was harvested from each stage (3T3-L1 preadipocytes, 2-day differentiated, 4-day differentiated, and 8-day differentiated adipocytes) using TRIzol reagent (Sigma-Aldrich, St. Louis, MO, USA) and subsequently used for RNA sequencing.

The mRNA from the prepared 3T3-L1 preadipocytes and adipocytes at different stages of differentiation was extracted and quantitatively isolated from total RNA using the poly(A) structure at the 3′ end of messenger RNA and related molecular biology techniques. The samples were subjected to a series of processes, including fragmentation, double-stranded cDNA synthesis, cDNA fragment modification, magnetic bead purification, fragment selection, and library amplification. Following the detection and quality control of the library, sequencing libraries suitable for the Illumina platform were obtained.

### 4.6. Differential Gene Expression Analysis

The differential expression analysis of the RNA-seq data was performed using the DESeq2 software (version 1.42.1, R 4.3.2) [46]. The preliminary phase entailed the creation of a DESeqDataSet object, which encompassed the initial count data, including the gene expression matrix and the pertinent experimental design information. The analysis used the DESeq function, which comprises data normalization, dispersion estimation, generalized linear model fitting, and statistical testing. The results were extracted via the results function, including the log2 fold change and the adjusted *p*-value. Genes were considered significantly differentially expressed if they exhibited an adjusted *p*-value of less than 0.05 and a log2FoldChange greater than 1. The generation of volcano plots and Venn diagrams was achieved by utilizing the “ggplot2” package and the VennDiagram package [47] to facilitate the visual representation of the outcomes.

### 4.7. Weighted Co-Expression Network Construction

A cluster of genes was constructed based on their expression profiles by implementing “WGCNA” [48]. For each pair of genes, an unsigned adjacency measure was calculated by raising the absolute value of their Pearson correlation coefficient to the power of the parameter β. The soft threshold β was set to 19 based on the scale-free topology fit index analysis, which indicated that this value would be optimal for the scale-free network. Subsequently, the Topological Overlap Measure (TOM) was calculated using the resulting adjacency matrix. TOM considers both direct relationships, as indicated by weighted pairwise correlations, and indirect relationships, as indicated by weighted correlations with other genes in the network. A hierarchical clustering analysis used the 1-TOM distance measure to identify gene modules.

For each module, the module eigengene (ME) was calculated, representing the first principal component of the gene expression matrix for that module. This provided a comprehensive overview of the module’s overall expression pattern. The Pearson correlation coefficient and associated *p*-value were calculated for each ME and PPT treatment to investigate the relationship between modules and phenotypes. The degree of gene connectivity was quantified by calculating the absolute value of the Pearson correlation coefficient. Genes exhibiting high intramodular connectivity (cor.geneModuleMembership > 0.8) and a robust correlation with PPT treatment (cor.geneTraitSignificance > 0.2) were identified as module eigengenes (MEs).

### 4.8. Gene Ontology and Pathway Enrichment Analysis

Gene Ontology (GO) and Kyoto Encyclopedia of Genes and Genomes (KEGG) pathway enrichment analyses were conducted on the selected genes using the clusterProfiler R package [49]. After the Benjamini–Hochberg correction, a GO enrichment analysis was performed for all three ontologies (biological process, cellular component, and molecular function) with a *p*-value cutoff of 0.05. Similarly, a Kyoto Encyclopedia of Genes and Genomes KEGG pathway enrichment analysis was conducted with a *p*-value cutoff of 0.05.

### 4.9. The Construction of a Protein–Protein Interaction Network

The STRING database (http://string-db.org/ (accessed on 28 May 2024)) analyzed protein interactions within the blue module. The construction and visualization of the protein–protein interaction (PPI) network was conducted using the Cytoscape software, version 3.8.0 (http://cytoscape.org/ (accessed on 28 May 2024)). Genes with a combined score of 0.9 or above derived from the STRING database were selected to construct the network model for Cytoscape visualization. In co-expression networks, the Maximal Clique Centrality (MCC) algorithm is the most effective method for identifying central nodes. Each node’s MCC was calculated using the CytoHubba plugin in Cytoscape. This study identified the ten genes with the highest MCC values as core genes. The STRING database (http://string-db.org/ (accessed on 28 May 2024)) was utilized for toy analyses between the MEs. Subsequently, the PPI network was constructed and visualized using the Cytoscape software, version 3.8.0 (http://cytoscape.org/ (accessed on 28 May 2024)).

### 4.10. Quantitative Real-Time RT-PCR

The total RNA samples were extracted using TRIzol reagent using the methodology previously described in the literature. Subsequently, the total RNA was reverse-transcribed into cDNA using an RT-qPCR kit (TransGen Biotech, Beijing, China). The PCR conditions were as follows: denaturation at 94 °C for 30 s, followed by 45 cycles of denaturation at 94 °C for 5 s, annealing at 60 °C for 15 s, and extension at 72 °C for 10 s. The relative expression levels of the target genes were calculated based on the cycle threshold (Ct) values as follows: ΔCt = Ct (target gene)-Ct (GAPDH); ΔΔCt = ΔCt (treatment) −ΔCt (control); Fold change = 2^−ΔΔCt^. The sequences of the primers used for the target genes are presented in Table 1.

### 4.11. Molecular Modeling

The 3D structure of protopanaxatriol was drawn using Chem3D, energy-optimized, and saved in a mol2 file. QPBQT files were exported using Autodock Tools 1.5.6. The receptor proteins PIKfyve (Protein Data Bank: 7k2v) and JAK1 (Protein Data Bank: 6dbn) were downloaded from the PDB (http://www.rcsb.org/ (accessed on 23 October 2024 )), and the docking center was localized using Discovery Studio Visualizer 2020 and the water molecules and original ligands. The edited receptors and ligands were imported into AutoDock Tool 1.5.6 to generate PDBQT files. Molecular docking was performed using the AutoDock program, and the results were visualized using Discovery Studio Visualizer 2020.

### 4.12. Statistical Analysis

GraphPad Prism 8.0 was used for statistical data analysis, and its standard deviation was used as the mean deviation (SEM). Three or more groups of gradual difference statistical analysis used one-way ANOVA. The Student *t*-test determined statistical significance except for the RNA-sequence analysis (Fisher exact test). *p* < 0.05 was considered as a statistically significant difference.

## 5. Conclusions

The differentiation of adipocytes is a complex and highly regulated biological process that involves the coordination of multiple signaling pathways and transcription factors. In this study, we employed the WGCNA method to identify sixteen gene modules significantly related to adipocyte differentiation. The core genes PIKfyve, STAT3, JAK1, CTTN, TYK2, JAK3, STAT2, STAT5b, SOCS3, and IRF9 are also considered essential candidate genes for regulating adipocyte differentiation. The results of this study suggest that PPT, an active ingredient in ginseng, may reduce lipid accumulation by negatively regulating the expression of genes such as PIKfyve, STAT3, and JAK1. Finally, molecular docking identified potential binding sites of PPT with PIKfyve and JAK1. The findings of this study provide new insights into the mechanism of adipocyte differentiation and offer important directions and critical targets for future research on the treatment of metabolic diseases.

## Figures and Tables

**Table 1:** Sequences of the primers for real-time RT-PCR.

Gene	Forward Primer (5′–3′)	Reverse Primer (5′–3′)
JAK1	GAAGTGATGGTGACTGGGAATCT	TTGTTCCACTCTTCCCGGATCTT
TYK2	ATGTCTGGTCCTTCGGGG TG	ATGAGATGATAGACCTCACAGGGAC
STAT3	CTCTTGGGACCTGGTGTGAATTA	GCTCCCGCTCCTTACTGATAAAG
JAK3	GACCCTCACTTCCTGCTGTATC	TGTCAGCAGGGATCTTGTGAAAT
STAT2	CAGTTGGCAGTTCTCCTCCTATG	ATGCGTCCATCATTCCAGAGATC
SOCS3	TCGCCACCTACTGAACCCTC	TGGTCCAGGAACTCCCGAAT
STAT5b	CACAGCTCCAGAACACGTATGA	CTGGTTGATCTGGAGGTGTTTCT
IRF9	GTGGTGCATGAGATCCAGGT	AGTGGGTCAGGTCTGGGAAA
PIKfyve	ATGGCCACAGATGACAAGAGTTCC	CAGACTGTGTTCTTGAAGGG
CTTN	TGGATAAGTCAGCTGTCGGC	TACTTGCCGCCAAAACCACT
GAPDH	AGAAGGCTGGGGCTCATTTG	AGGGGCCATCCACAGTCTTC

## Data Availability

Data supporting the results of this study are available from the corresponding author upon request.

## Supplementary Materials

The following supporting information can be downloaded at: https://www.mdpi.com/article/10.3390/ijms252212254/s1.

## Abbreviations

PPT	Protopanaxatriol
WGCNA	Weighted Gene Co-expression Network Analysis
PIKfyve	1-phosphatidylinositol-3-phosphate 5-kinase
STAT3	Signal transducer and activator of transcription 3
JAK1	Janus kinase 1
CTTN	Src substrate cortactin
TYK2	Non-receptor tyrosine-protein kinase
INS	Insulin
IBMX	3-Isobutyl-1-methylxanthine
KEGG	Kyoto Encyclopedia of Genes and Genomes
PPI	Protein–protein interaction
CCK-8	Cell Counting Kit-8
GS	Gene significance
JAK3	Janus Kinase 3
STAT2	Signal transducer and activator of transcription 2
STAT5b	Signal transducer and activator of transcription 5b
SOCS3	Suppressor of cytokine signaling 3
IRF9	Interferon regulatory factor9
NF-κB	Nuclear factor-ĸB
DMEM	Dulbecco’s modified Eagle’s medium
GO	Gene Ontology
DEX	Dexamethasone
MEs	Module eigengenes
FBS	Fetal bovine serum
PDQ	Pseudo-ginsenoside DQ
MM	Module membership
