# Supplementary material for: Weighted Gene Co-Expression Network Based on Transcriptomics: Unravelling the Differentiation Dynamics of 3T3-L1 Preadipocytes and the Regulatory Mechanism of Protopanaxatriol
Source: Int J Mol Sci. 2024 Nov 14;25(22):12254. doi: 10.3390/ijms252212254 (PMC11594308; doi:10.3390/ijms252212254)
Supplement: Supplementary file 1 [file ijms-25-12254-s001.zip › ijms-3259942-supplementary.pdf]

Supporting Information

Weighted gene co-expression network based on transcriptomics: Unravelling the differentiation dynamics of 3T3-L1 preadipocytes and the regulatory mechanism of protopanaxatriol

Yaru Zhao a,b,1, Xv Wang a,b,1, Hongbo Teng a,b,1, Tianyi Zhao b,1, Wendyam Marie Christelle Nadembega b, Xinhua Fan a,b, Wenxin Zhang a,b, Bowen Fan a,b, Yuye Chi a, Yan Zhao a,b,* and Shuangli Liu a,b,**

a College of Chinese Medicinal Materials, Jilin Agricultural University, Changchun, Jilin Province, China

b International Joint Laboratory for Development of Animal and Plant Resources for Food and Medicine, Changchun, Jilin Province, China

*Correspondence: Corresponding author at: Xincheng Street 2888, College of Chinese Medicinal Materials, Jilin Agricultural University, Changchun 130118, Jilin, China.

E-mail addresses: zhaoyan@jlau.edu.cn (Y. Zhao), liu-shuangli@163.com (S. Liu).

1 Co-first author

Supplementary Figure S1A:

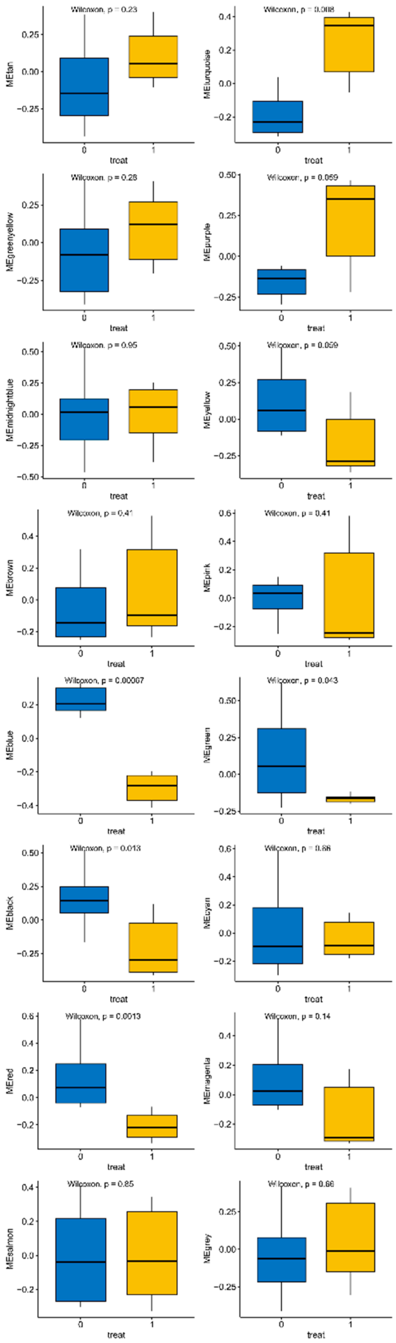

Supplementary Figure S1A:

Box plot illustrating the correlation between modules and phenotypic characteristics. The x-axis depicts the sample information, while the y-axis represents the module eigenvalues.
